# Range of motion in the cervical spine after odontoid fracture treated with anterior screw fixation

**DOI:** 10.1186/s13018-019-1135-8

**Published:** 2019-04-15

**Authors:** Andżelina Wolan-Nieroda, Andrzej Maciejczak, Agnieszka Guzik, Grzegorz Przysada, Ewa Szeliga, Mariusz Drużbicki

**Affiliations:** 1 Medical Faculty, University of Rzeszów, Kopisto 2A Avenue, 35-959 Rzeszow, Poland; 2 Department of Neurosurgery, St Luke Hospital, Lwowska 178 Street, 33-100 Tarnow, Poland; 3 Institute of Physiotherapy, University of Rzeszow, Warszawska 26 a, 35-205 Rzeszów, Poland

**Keywords:** Range of motion, Odontoid fracture, Cervical spine, Cervical collar, Pain

## Abstract

**Background:**

It is believed that direct odontoid screw fixation preserves the physiological cervical range of motion following surgery. However, there are no clinical studies confirming the motion sparing value of this technique.

This study aims to (1) to assess active cervical range of motion following types II and III odontoid fracture, successfully treated with anterior odontoid screw fixation, and (2) to examine the relationship between the range of motion of the head and duration of collar usage, neck pain, quality of life, and patients’ age.

**Methods:**

The study involved 41 patients subjected to a procedure of direct osteosynthesis of the dens with lag screw. Following the operation all the patients had to wear a cervical collar to protect the osteosynthesis. The control group consisted of 41 individuals with no clinical diagnosis of any cervical spine disorders. The spinal motion was assessed using multi-cervical unit, taking into account bending/extension, left and right lateral flexion, and left and right axial rotation.

**Results:**

In the study group, spine mobility correlated with the duration of hard collar usage following the operation, with a longer duration corresponding to poorer spine mobility at the end of the treatment. Statistically significant correlation was observed in the case of extension (*p* < 0.021) and axial rotation (*p* < 0.007). In the study group, there was a negative correlation between the range of motion and the patients’ age, i.e., the older the patient the poorer his/her spinal mobility (*p* < 0.001).

**Conclusions:**

Active cervical range of motion in patients following direct osteosynthesis of the dens, augmented with a hard collar, was significantly lower than in the control population, and it correlated negatively with the duration of collar usage, the patients’ age, and intensity of spinal pain.

## Introduction

From the viewpoint of spinal kinematics, direct osteosynthesis is the most effective method of surgical treatment of odontoid fractures. It is believed that, unlike posterior atlantoaxial fixation, the method preserves physiological range of motion in the upper cervical spine, which is responsible for nearly half of the entire range of motion of the head relative to the torso [1–3]. In the literature, there are no clinical studies focusing on cervical mobility in patients following anterior odontoid screw fixation or conservative treatment involving immobilization in external hard collar or in halo vest. Meanwhile our own clinical practice provided anecdotal evidence against motion sparing value of direct anterior odontoid osteosynthesis. Therefore, we decided to carry out a clinical study in order to verify this anecdotal evidence. The initial findings of this research have already been published [4, 5]. The present study reports ranges of motion of the head in patients following direct odontoid screw fixation.

## Material and method

### Study group

The analyses took into account patients recruited from among 214 individuals with odontoid process fracture, receiving treatment in a neurosurgery ward of a large regional hospital during 2004–2012. To qualify for the study, the patients had to meet the following criteria:

- Isolated acute axis fracture, without co-existing fractures in the upper or subaxial section of the cervical spine;
- Type II or III odontoid fracture;
- Direct anterior osteosynthesis of the dens with lag screw;
- Completed osseous union of the dens, documented with computed tomography (CT) scan;
- No cervical spine kinesiotherapy was administered following the operation to improve cervical mobility.

Fractures which occurred less than 3 weeks earlier were classified as acute. In all the cases, anterior single lag screw fixation was performed. The placement of the screw in the body and dens of the axis was verified with CT scan. Following the operation, all the subjects were provided with hard cervical collars, for the duration of the healing process. Fracture healing was inspected via CT bone windows in three planes (transverse, sagittal, and frontal). It was assumed that bone union had occurred if there were visible osseous bridges in at least one of the three reconstructed planes. The first CT scan assessing fracture healing was performed not earlier than 2 months after the operation. If that scan showed no radiologic features of the bone union, subsequent scans were performed every 2 months, until fracture healing was completed. The subjects in the study group used hard collars for a minimum of 2 months and for a maximum of 8 months.

Ultimately, the study group consisted of 41 patients meeting the inclusion criteria (15 females and 26 males). The mean age of the subjects was 49.2 ± 18.3 years (ranging from 18 to 80).

### Control group

It consisted of 41 subjects (22 females and 19 males) without clinically diagnosed disorders of or self-reported problems connected with the cervical spine. The controls were matched for age to the study group. On average, they were 48.6 ± 17.5 years old (ranging from 19 to 79).

## Measurements of spinal range of motion

**Fig. 1:**
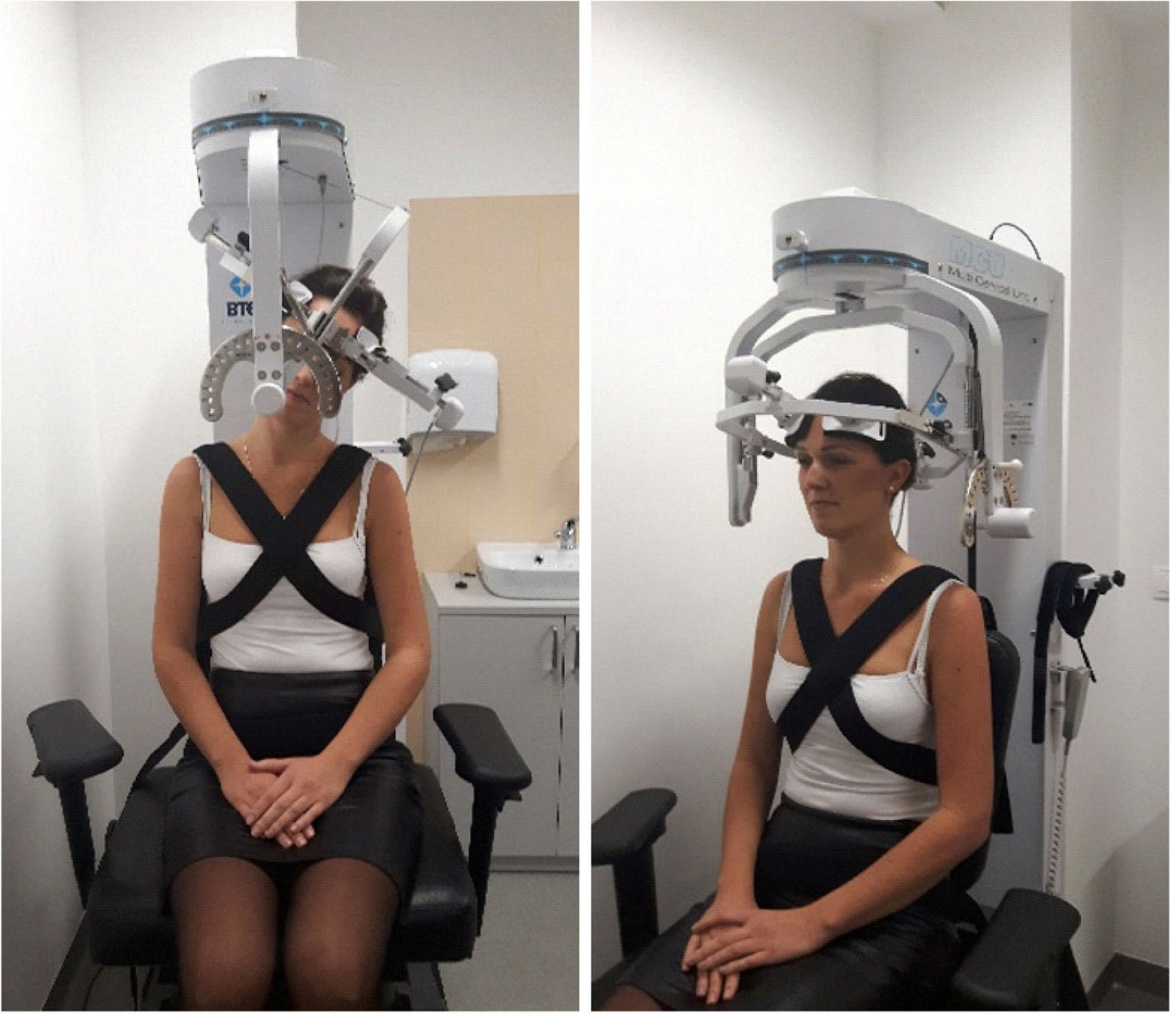
Measurement of the range of cervical spine motion using MCU (multi-cervical unit) device

The range of cervical motion was measured using multi-cervical unit, following the manufacturer’s instruction (Fig. 1). The measurements were performed by two highly qualified physiotherapists, each with over 10-year professional experience in management of spinal dysfunctions. One physiotherapist examined the controls and the other physiotherapist performed the measurements in the study group, without contacting each other.

The measurements were performed to assess the range of forward bending and extension, left and right lateral flexion, and left and right axial rotation. The ranges of motion in the study group were compared to the ranges of motion in the controls.

Neck pain was assessed using visual analogue scale (VAS).

## Statistical analysis

Statistical analyses of the data were computed using Statistica 10.0 software. The consistency of the distributions with the normal distribution was verified with Shapiro–Wilk *W* test and homogeneity of variance was assessed with Levine’s test.

Comparison of the cervical ranges of motion identified in the study group and in the controls was carried out using *z* test for two independent samples.

The correlations of spinal range of motion and (i) patients’ age, (ii) duration of collar usage, and (iii) neck pain were calculated using Spearman’s rank correlation coefficient*.* Statistical significance was assumed at *p* < 0.05.

## Results

### Range of motion of the head: the study group versus the control group

The ranges of motion in the study group were lower than in the controls in all the three planes, with the greatest difference observed in extension (Table 1). All the differences in the ranges of movement were statistically significant.

**Table 1:** Cervical range of motion in the study group and in the controls

Movement	Study group (*n* = 41)			Control group (*n* = 41)			*p*	
	$\overline{x}$	Me	SD	$\overline{x}$	Me	SD	*t*/*Z*	*p*
Bending forward	39.71	40.00	16.77	54.59	52.00	12.20	− 4.08	< 0.001
Extension	42.76	40.00	20.59	71.88	70.00	14.90	− 5.59	< 0.001
Left lateral flexion	26.51	25.00	14.27	42.54	40.00	9.75	− 5.04	< 0.001
Right lateral flexion	26.54	20.00	14.46	42.83	40.00	8.95	− 5.07	< 0.001
Left rotation	43.71	50.00	21.42	66.56	70.00	12.78	− 4.75	< 0.001
Right rotation	45.76	50.00	23.32	66.66	70.00	13.11	− 5.00	< 0.001

*t* result in Student’s *t* test for independent variables; *Z* result in Mann–Whitney *U* test; *p* probability level

### Correlation of cervical range of motion and (i) duration of hard collar usage following the operation, (ii) intensity of neck pain, and (iii) age

(i) Spine mobility versus duration of collar usage following operation.

Duration of collar usage was related to cervical range of motion. The patients who used a collar for 6 months or more presented lower ranges of motion than those wearing a collar for a duration of 2–5 months. However, the differences were not significant, except for the movement of extension (Table 2).

**Table 2:** Cervical range of motion versus duration of hard collar usage

Movement	3–5 months (*n* = 13)			> 6 months (*n* = 27)			*p*	
	$\overline{x}$	Me	SD	$\overline{x}$	Me	SD	*t/U*	*p*
Bending forward	40.50	44.00	17.23	39.30	40.00	16.84	185.0	0.924
Extension	53.00	60.00	17.00	37.44	40.00	20.54	110.5	0.030
Left lateral flexion	31.79	25.00	18.25	23.78	25.00	11.13	141.5	0.194
Right lateral flexion	30.86	25.00	19.34	24.30	20.00	10.92	155.0	0.362
Left rotation	52.50	52.50	18.89	39.15	40.00	21.55	127.5	0.091
Right rotation	55.71	45.00	21.11	40.59	50.00	23.08	122.5	0.067

*t* result in Student’s *t* test for independent variables; *U* result in Mann–Whitney *U* test; *p* probability level

Spearman rank correlation showed that the range of spine mobility correlated negatively with the duration of collar usage, where lower duration of collar usage corresponded with greater range of motion of the head. The correlation, however, was weak, and it was only statistically significant in the case of axial rotation and extension. The strongest correlation was found in the case of right rotation, *p* < 0.007, and slightly weaker in the case of extension, *p* < 0.02 and left rotation, *p* < 0.03 (Table 3).

(ii) Cervical spine mobility versus neck pain.

**Table 3:** Correlation between duration of hard collar usage and cervical range of motion

Duration of cervical collar usage versus:	*R*	*p*
Bending forward	− 0.18	0.269
Extension	− 0.36	0.021
Left lateral flexion	− 0.22	0.165
Right lateral flexion	− 0.07	0.667
Left rotation	− 0.34	0.030
Right rotation	− 0.41	0.007

*R* result in Spearman’s rank correlation test; *p* probability level

**Fig. 2:**
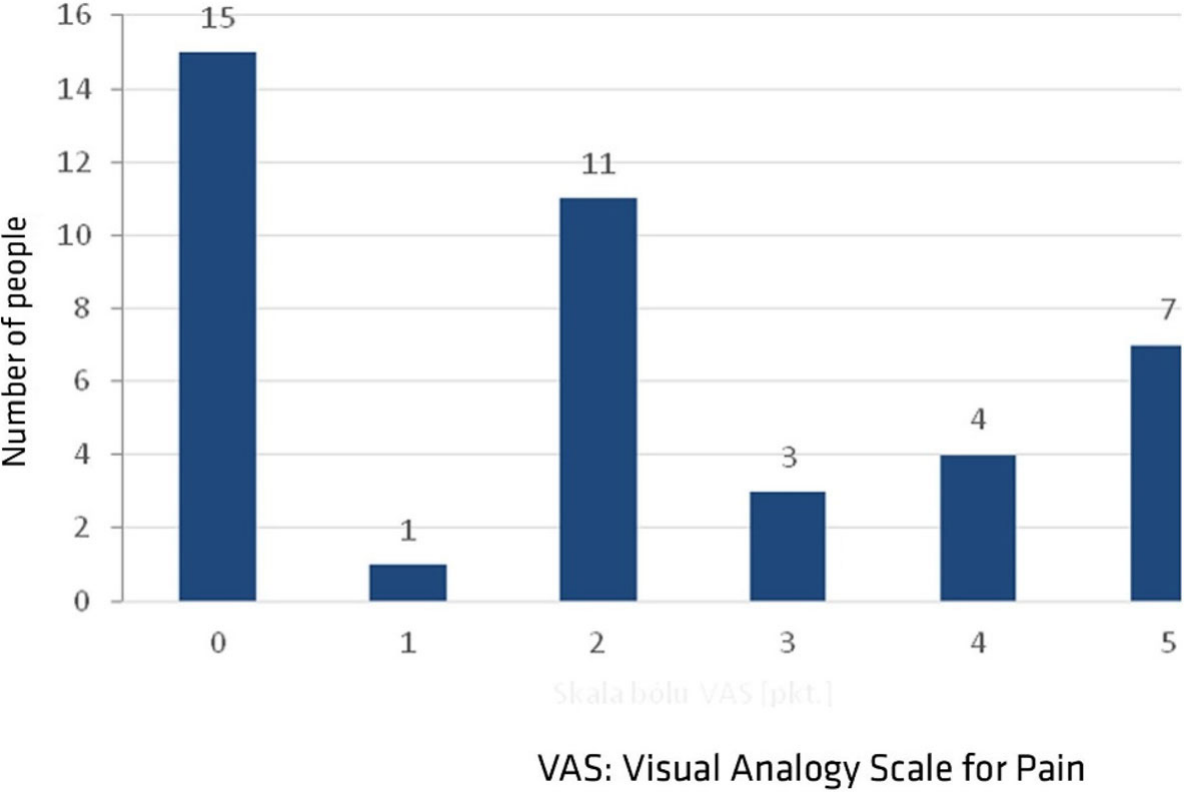
Pain intensity according to the VAS scale

Neck pain was reported by 26 (63.4%) subjects in the study group. Mean pain intensity amounted to 2.02 ± 1.89. In assessing pain intensity a vast majority of the patients used rating 2 or higher, and 5 points was the highest rating of pain intensity on VAS (Fig. 2). The range of motion moderately correlated with pain intensity in the case of lateral flexion, and greater neck pain corresponded with lower range of lateral flexion. This correlation was statistically significant. On the other hand, the correlations between pain intensity and ranges of motion in the remaining planes were weak or mild, with no statistical significance (Table 4).

(iii) Cervical spine mobility versus age.

**Table 4:** Correlations between cervical range of motion and neck pain

Pain versus:	*R*	*p*
Bending forward	− 0.30	0.054
Extension	− 0.21	0.191
Left lateral flexion	− 0.43	0.005
Right lateral flexion	− 0.42	0.006
Left rotation	− 0.07	0.680
Right rotation	− 0.16	0.322

*R* result in Spearman’s rank correlation test; *p* probability level

Spinal mobility negatively correlated with the subjects’ age; the older the patient, the lower range of motion could be observed. The strongest correlation was identified in the case of left lateral flexion and right rotation at *p* < 0.001 (Table 5).

**Table 5:** Correlation of cervical range of motion and age

Age versus:	*R*	*p*
Bending forward	− 0.33	0.036
Extension	− 0.32	0.042
Left lateral flexion	− 0.61	0.001
Right lateral flexion	− 0.46	0.003
Left rotation	− 0.36	0.020
Right rotation	− 0.50	0.001

*R* size of correlation, *p* significance level, maximum probability of error

## Discussion

Our personal clinical practice suggested that following both selective odontoid screw fixation and conservative treatment of odontoid fracture, patients may experience severe limitations in cervical spine mobility. In the literature, there are no research reports related to this issue. Theoretically, treatment based on both of these methods should preserve the full range of spinal motion. The present findings, however, appear to challenge to the above opinion. There are a few possible explanations for this phenomenon. Firstly, limited cervical spine mobility may be caused by long-term immobilization in hard collar. Immobilization is used not only in conservative treatment of fractures but also following odontoid osteosynthesis, in order to prevent osteosynthesis failure before bone union is achieved. Long-lasting immobilization may lead to contractions of capsular ligaments in the entire cervical spine, in particular in the C1/C2 segment where the structure is well-developed and plays an important role in stabilizing the spine.

Selective limitation of mobility in the C1/C2 region may be related to fibrous scar tissue in the area of capsular ligament of the median atlantoaxial joint and/or the lateral atlantoaxial joint. The problem could particularly be magnified by significant atlantoaxial dislocation which may lead to rupture of articular capsules in lateral atlantoaxial joints and to injury of certain ligaments in this area. In the case of ligament injuries, the recovery process may involve formation of rigid, non-flexible scar reducing the range of motion between the atlas and the axis. Other factors leading to reduced mobility include post-traumatic neck pain, which forces the patient to limit head movements, as well as a lack of rehabilitation after the healing process of odontoid fracture has been completed.

Our study presents evidence confirming limited cervical spine mobility in patients following complete odontoid fracture healing. Compared to the controls, the patients with odontoid fracture had lower ranges of motion in all the planes.

Immobilization of cervical spine in a hard collar is the basic element of conservative treatment following odontoid fracture or it is applied as an aid supporting the process of osteosynthesis [4–6]. In both cases, it can be expected there will be consequences of long-term immobilization of the cervical spine in a hard collar, such as contractions of capsular ligaments in the spine. Similar effects can be produced by the use of halo vests [7–9].

Notably, in conservative treatment, there is a tendency to use hard collars rather than halo vests [4–6]. For types I and III fractures, this type of orthosis does not raise too much controversy and discussion, as the rates of fusion achieved using this treatment strategy are considered acceptable [7–12]. The reported union rates are 100% for type I, and over 85% for type III [13–15].

We have not found any research reports discussing mobility of the cervical spine and head following conservative treatment of odontoid fractures. Our findings suggest that immobilization of the cervical spine using card collar may lead to reduced cervical mobility. All the patients from our series of odontoid screw fixation cases wore hard collars until complete union was achieved. The question, however, arises whether such limitations in the range of motion of head would have appeared if our patients had not been subjected to immobilization during postoperative period and had been allowed to move freely their heads immediately after dens osteosynthesis. Unfortunately, we do not have a control group of odontoid osteosynthesis without postoperative immobilization. We routinely applied hard collar postoperatively in every patient undergoing direct osteosynthesis of the dens. We believe this policy increases the chance of union and prevents screw migration or destabilization of osteosynthesis. We recognize that majority of researchers investigated patients without external immobilization after odontoid screw fixation. So in fact, we cannot clearly conclude whether limitations in the range of motion after direct osteosynthesis of the odontoid process is a result of the osteosynthesis itself or external immobilization in a hard collar or both. Our study, however, provides evidence that the former or the latter or both may lead to limitations in head movements. And we believe this is an important finding of our study. We hope we will soon present results showing cervical mobility in patients following odontoid screw fixation without external immobilization, and we will compare these findings with the range of motion of patients with odontoid osteosynthesis and postoperative external immobilization.

Limitations in cervical spine mobility, experienced by patients with odontoid fracture, appear to be an underestimated problem. It would seem reasonable to ensure routine tests assessing the range of motion of the head in these patients following complete fracture healing. This means that completed union does not necessarily define the end of treatment, since these patients may need rehabilitation designed to restore the maximum possible cervical spine mobility.

## Conclusions

Patients with completely healed odontoid fracture, following direct osteosynthesis of the dens augmented postoperatively with a hard collar, present lower neck/head mobility than control asymptomatic group. The range of motion of the head in the study group correlates negatively with age, neck pain, and duration of hard collar usage.

## Abbreviations

MCU: Multi-cervical unit
VAS: Visual analogue scale
